# Oral and transdermal administration of lipopolysaccharide safely enhances self-healing ability through the macrophage network

**DOI:** 10.3389/fimmu.2025.1563484

**Published:** 2025-03-31

**Authors:** Gen-Ichiro Soma, Masataka Oda, Vindy Tjendana Tjhin, Chie Kohchi, Hiroyuki Inagawa

**Affiliations:** ^1^ Control of Innate Immunity, Technology Research Association, Takamatsu, Kagawa, Japan; ^2^ Research Institute for Healthy Living, Niigata University of Pharmacy and Applied Life Sciences, Niigata, Japan

**Keywords:** lipopolysaccharide, macrophage, macrophage network, innate immunity, self-healing ability

## Abstract

Lipopolysaccharide (LPS), also known as an endotoxin, is derived from Gram-negative bacteria. The intravenous administration of LPS induces an inflammatory response and causes systemic inflammation, such as cytokine storm. Gram-negative bacteria that produce LPS are found in the environment and digestive tract. The mucous membrane, the primary barrier between the interior of the body and the external environment, is constantly exposed to LPS. Moreover, no toxicity is observed when administering LPS through the mucous membranes of the mouth or skin. The presence of LPS in the mucous membranes is necessary not only for maintaining health but also for inducing preventive and therapeutic effects against multiple diseases when administered orally or topically. LPS is an environmental substance that is useful when administered to mucous membranes. The general information emphasizes the role of LPS as an inflammatory substance that occurs when administered intravenously. Therefore, the valuable role of LPS is unknown. Thus, mucosal administration of LPS has received little attention, and the mechanism underlying the expression of its beneficial effects has not been fully elucidated. We proposed a comprehensive concept, the “macrophage network,” which proposes a regulatory system in which the mucosa receives environmental information, membrane-bound cytokines are expressed in phagocytes (macrophages), and these macrophages migrate distally to exert effects, such as anti-inflammatory and tissue repair effects, on distal tissues through cell-to-cell communication (juxtacrine signaling) with tissue macrophages. This macrophage network is effective not only for preventing and treating diseases but also for increasing the efficacy of pharmaceuticals. This review aims to investigate the preventive and therapeutic effects of oral and transdermal administration of LPS on various diseases and present an introduction to the concept of the macrophage network and the latest findings.

## 1 Introduction

Living organisms must maintain homeostasis and survive despite being exposed to diverse environmental changes. They must eliminate invading pathogens, such as viruses and bacteria, and tissue damage caused by other factors, such as ultraviolet rays, chemicals, and heat. Living organisms have developed mechanisms to maintain homeostasis by actively using environmental information that is useful for survival. In the context of immune responses, the primary reception of this information is mainly carried out by innate immunity. Phagocytes, the main cells responsible for innate immunity, not only identify and eliminate foreign invaders, such as bacteria and viruses, but also respond to a wide range of other environmental stimuli, such as heat, pressure, and low-molecular-weight substances, such as adenosine and salt, and play a significant role in maintaining the homeostasis of the individual.

Until recently, little attention has been paid to the biological responses to these everyday environmental stimuli to maintain homeostasis. This may be due to the difficulty of extracting the necessary information from miscellaneous environmental information and of understanding how an individual organism maintains homeostasis even when this information is extracted. Innate immunity is a well-understood mechanism for maintaining homeostasis. The research focusing on the identification of lipopolysaccharide (LPS) in the outer membrane of Gram-negative bacteria and its receptor, Toll-like receptor 4 (TLR4), has dramatically advanced our understanding of the molecular level of innate immunity involving macrophages, dendritic cells, neutrophils, epithelial cells, etc. (1–3). LPS is a substance that activates these natural immune system cells in minute quantities (4) and is well known to induce systemic inflammation when administered intravascularly, even at low doses (5). We focused on innate immunity and searched for environmental factors that control macrophages via the mucosa. We found that mucosal administration of LPS is highly useful as a molecule that controls innate immunity (6). The administration of LPS via mucosal routes, such as oral or transdermal delivery, not only exhibits no toxicity but also, contrary to conventional understanding, demonstrates anti-inflammatory effects and tissue repair and regeneration capabilities. The mechanism by which LPS contributes to the maintenance of systemic homeostasis via mucosal innate immunity is still largely unknown. To elucidate this mechanism, we have focused on macrophages, which maintain tissue homeostasis, and have hypothesized that a regulatory system called the “macrophage network” (7, 8). Additionally, *in vitro* test systems using cultured cells are another factor that hinders our understanding of the physiological effects of LPS. Until now, LPS has been used as an inflammatory agent by stimulating monocyte-derived macrophage cell cultures or bone marrow-derived cells at high concentrations (≥100 ng/ml) or by injecting it into the abdominal cavity or blood vessels of animals (9). This model could be a useful tool for treating sepsis and inflammatory diseases. However, LPS is naturally present in the skin, intestinal tract, and respiratory tract, and it cannot be treated the same way as in the injection model. It has become clear that LPS has useful physiological activity when administered orally or topically and has a previously unknown homeostatic maintenance effect (10).

This review aims to demonstrate that oral and transdermal administration of LPS is beneficial for preventing and treating chronic inflammatory diseases, such as allergies and dementia, which are difficult to treat with current medical treatments. Additionally, this review discusses the role of LPS as an environmental substance in maintaining homeostasis in individuals via the mucosal membrane and the macrophage network hypothesis.

## 2 Self-healing, physiological inflammation, and innate immunity

Innate self-healing ability is a critical physiological mechanism for maintaining individual health. Its role in eliminating exogenous agents, such as viruses and bacteria, remains a key research area. At sites of foreign invasion, tissue-resident macrophages initiate the molecular recognition of pathogens through pathogen-associated molecular pattern recognition receptors, including TLRs, Nod-like receptors, C-type lectin receptors, and RIG-I-like receptors. The activation of these pattern recognition receptors triggers macrophage-mediated responses, including the recruitment of neutrophils and monocytes, generation of reactive oxygen species (ROS), and production of chemokines and cytokines (11). These events manifest as inflammatory symptoms, such as edema, erythema, fever, and pain, which are traditionally viewed under pathological conditions (12).

Inflammation has been increasingly recognized as a physiological process that plays an important role in tissue repair and healing. During immune challenges, such as injury and infection, tissue macrophages recognize pathogens and initiate an acute inflammatory response. This process involves tissue destruction via ROS production to eliminate the offending agent, followed by a highly regulated process of tissue repair and regeneration. The repair phase involves debris phagocytosis, angiogenesis, and tissue remodeling (13). The concept of physiological inflammation was first proposed by Metchnikoff, who asserted its essential role in maintaining homeostasis through its dual function of pathogen clearance and tissue repair (14, 15). As key mediators of innate immunity, macrophages play an important role in the inflammatory process, participating from its onset to resolution (13).

The theories of Metchnikoff on physiological inflammation are being revisited, extending the role of innate immunity beyond classical inflammation to encompass broader self-healing mechanisms (16). Initially, innate immunity was understood as a defense system that recognizes and neutralizes exogenous pathogens, such as bacteria, viruses, and fungi (17). Today, it is considered a frontline mechanism capable of eliminating endogenous aberrant elements, such as senescent or apoptotic cells, denatured proteins, and oxidized lipids, which disrupt homeostasis (18–21). This orchestrated process, which is referred to as physiological inflammation, enables the clearance and repair of damaged tissues, thereby promoting recovery (22).

Physiological inflammation is particularly related to scenarios in which tissue damage is caused by pathogens, aging, or malignancies. Macrophage-mediated processes clear damaged cells and tissues, facilitating their repair and regeneration (18). Thus, innate immunity is primarily driven by macrophages and is the cornerstone of self-healing.

Failure to effectively eliminate pathological stimuli through the physiological inflammatory process may lead to the accumulation of foreign substances and sustained cellular damage, thereby leading to chronic inflammation and impaired tissue function and homeostasis. Therefore, the decline in innate immune function compromises the recognition and resolution of internal and external aberrations, ultimately reducing the organism’s self-healing ability.

## 3 Strengthening self-healing through activation of innate immunity

Significant attention has been paid to the role of innate immunity in infection prevention, particularly during the spread of novel coronaviruses (23). Evidence suggests that the Bacillus Calmette–Guérin (BCG) vaccine promotes a rapid immune response to COVID-19, which potentially mitigates disease progression through the early suppression of viral replication mediated by interferon-alpha (IFN-α) induction (24). Furthermore, the BCG vaccine enhances IFN-α production during viral entry through epigenetic modifications in macrophages (25). This durable infection-preventive effect, known as “trained immunity” or “innate immune memory,” indicates the enduring nature of such responses (26). Therefore, the maintenance of robust innate immune functionality is increasingly recognized as an important strategy for preventing and treating infectious diseases.

The prevalence of lifestyle-related diseases, such as cancer, diabetes, and Alzheimer’s disease, has increased over the past decade (27–29). Although these conditions are associated with lifestyle factors, such as physical inactivity and excessive consumption of high-calorie diets, aging also plays a critical role. Aging is associated with decreased macrophage phagocytic activity, reduced clearance of foreign substances, and chronic inflammation, which is driven by phenotypic shifts from tissue-repairing M2 macrophages to proinflammatory M1 macrophages (30). Additionally, disruption of homeostatic mechanisms can exacerbate these conditions (30, 31). As proposed in the “hygiene hypothesis,” modern urban lifestyles reduce exposure to symbiotic microbiota and innate immune stimuli, such as LPS. This reduction alters the balance of acquired immunity, thereby increasing the prevalence of allergic diseases (32–34). These observations underscore the importance of innate immunity in maintaining physiological homeostasis.

Macrophages are heterogeneous immune cells with high plasticity that adapt to environmental cues and exhibit distinct functional phenotypes. M1 macrophages are responsible for the clearance of pathogens through the production of inflammatory cytokines, chemokines, and ROS. In contrast, M2 macrophages mediate anti-inflammatory responses and promote tissue repair and regeneration through the secretion of anti-inflammatory cytokines and growth factors (13, 35–40). Recent therapeutic approaches targeting M2 macrophages have shown promise in managing Alzheimer’s disease (41), maintaining skin health (18, 42), restoring mobility after spinal cord injury, and treating amyotrophic lateral sclerosis and liver cirrhosis (43–48). Schwartz et al. reported that inducing tissue-reparative macrophages can regulate physiological inflammation and provide therapeutic potential for traditionally challenging conditions, such as spinal cord injuries (49, 50).

Therefore, the activation of innate immunity plays an important role not only in preventing infection but also in strengthening self-healing mechanisms across various diseases, including lifestyle-related conditions. This perspective provides valuable insights into the scientific exploration of self-healing, an essential and broad concept in health maintenance and disease management.

## 4 Innate immune regulation by macrophage networks

**Figure 1:**
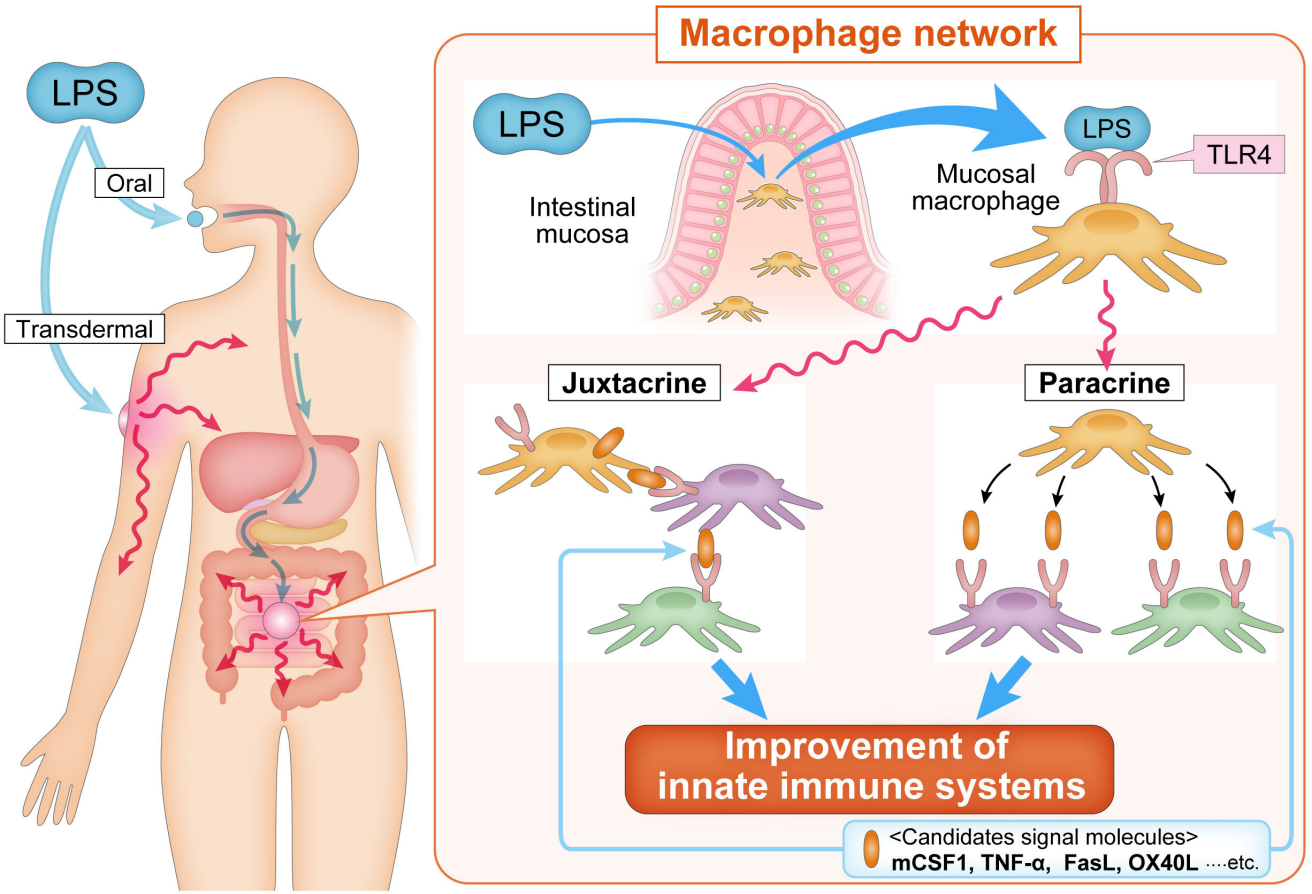
The macrophage network concept. The macrophage network hypothesis is based on the idea that the transmission of information is mediated by secreted cytokines (paracrine) and/or by cell-to-cell signaling (juxtacrine) via membrane-bound cytokines expressed on macrophages. Oral and transdermal administration of LPS activates macrophages of the intestinal mucosa, which induce the activation of systemic macrophages in a paracrine manner and/or membrane-bound cytokine-expressing macrophages migrate to tissues and communicate with systemic macrophages in a juxtacrine manner. mCSF1, TNF-α, FasL, OX40L, and other signaling molecules have been proposed to be involved in propagating the activation of mucosal macrophage to systemic macrophage. FasL, Fas ligand; LPS, lipopolysaccharide; mCSF1, membrane-bound colony stimulating factor-1; OX40L, OX40 ligand; TLR4, toll-like receptor 4; TNF-α, tumor necrosis factor-alpha.

Macrophages are widely distributed across tissues and play central roles in immune responses and homeostasis. Macrophages constitute approximately 50% of the total immune cell mass in the human body (51), highlighting their significant importance in immune defense mechanisms. Tiemeijer et al. reported that macrophage interactions vary according to density and the environmental context, revealing heterogeneity at the single-cell level (52). This quantitative and qualitative diversity indicates that macrophages significantly contribute to the maintenance of organismal homeostasis. However, the mechanism underlying macrophage-mediated homeostatic regulation is not fully elucidated. Tissue macrophages are increasingly recognized as integral to the clearance of dead cells, cellular debris, immune complexes, bacteria, and other waste products. Additionally, they play an essential role in organ development and tissue homeostasis (31). Crosstalk between tissue-resident macrophages and local cells has been reported in the heart (53) and adipose tissue (54), where mitochondria serve as signaling intermediaries. In addition to their tissue-specific roles, macrophages are believed to function as part of a systemic network (**Figure 1**). This “macrophage network” hypothesis posits that macrophages in specific tissues communicate with counterparts in distant tissues to regulate homeostasis (8). Recent studies have suggested that this macrophage network operates through integrin-mediated motor control and inter-macrophage communication, a mechanism observed even in primitive multicellular organisms, such as sponges, where migratory macrophage-like cells maintain organismal integrity in the absence of the nervous system (55, 56). Macrophage-mediated signaling relies on cytokines. Information transmission occurs through paracrine or juxtacrine signaling, in which membrane-expressed cytokines, such as tumor necrosis factor (TNF), interleukin-1 alpha, Fas ligand, TNF-related apoptosis-inducing ligand, stem cell factor, and transforming growth factor alpha, facilitate direct communication between macrophages and target cells (57). Examples of juxtacrine signaling include tumor necrosis factor (TNF) (58), IL-1 alpha (59), Fas ligand (60), TRAIL (61), SCF (62), and TGF-alpha (63).

Activated macrophages in one tissue may migrate to distal tissues, thereby transmitting information to local cellular populations. Migratory macrophages serve as critical mediators of systemic immune regulation in higher organisms. The systemic signaling capacity of macrophages is highlighted in several examples. During cardiac stress, such as exercise, cardiac macrophages are activated by colony-stimulating factor 2 (CSF2) secreted by renal macrophages, facilitating cardiomyocyte proliferation (64). Similarly, oral administration of LPS induces the expression of membrane-bound CSF1 in peripheral leukocytes, enhancing the phagocytic capacity of microglia in the brain and converting them into neuroprotective M2-type macrophages (65). In the gastrointestinal system, LPS activates intestinal macrophages to induce bone morphogenetic protein-2, which stimulates enteric neurons and enhances peristalsis, whereas neurons secrete CSF2 to promote macrophage proliferation (66). These processes are mediated by TLR4 ligands derived from the gut microbiota (67). These findings support the existence of a tissue macrophage-mediated signaling system, suggesting that a functional macrophage network extends across multiple organ systems (68–72). In Western medicine, drug delivery systems target local areas to enhance therapeutic efficacy. However, environmental stimuli, such as those received by the skin and mucosa, often affect distant tissues. Similar phenomena have been observed in adaptive immunity, wherein dendritic cells and monocytes transport pathogen antigens from the site of infection or vaccination to the lymph nodes, activating CD4+ and CD8+ T cells in lymphoid tissues (73). The diverse population of tissue macrophages that can receive environmental signals and transmit them across the body supports the hypothesis that the macrophage network plays a pivotal role in maintaining systemic health. Therefore, macrophages act as a highly versatile network that integrates environmental signals and orchestrates immune responses across tissues. The macrophage network is a critical component of innate immune regulation and systemic homeostasis, indicating its potential as a therapeutic target for promoting health and treating diseases.

## 5 Regulation of innate immunity by LPS as an environmental agent

Improvements in healthcare access, nutrition, and sanitation have contributed to increased longevity in developed countries. However, the incidence of allergies, lifestyle-related diseases, emerging infectious diseases, and cancers that are challenging to treat is increasing in modern societies (74–77). This is often attributed to enhanced sanitation in urban environments and the overuse of antibiotics, which lead to a decline in the natural functionality of the immune system (34, 78, 79). LPS, as a potential missing environmental factor, has emerged as a focus of interest for compensating for diminished innate immune function.

LPS is a major component of the outer membrane of Gram-negative bacteria. It is a macromolecule with a molecular weight of approximately 5–100 kDa. It has three main components: lipid A, core polysaccharide, and O-antigen. The biological activity of LPS is primarily mediated by its interaction with TLR4. LPS can be categorized based on the bacterial source into lipid A with six to seven fatty acids (e.g., Gammaproteobacteria) and lipid A with four to five fatty acids (e.g., *Bacteroides*). The former exhibits a 100-fold greater binding affinity for TLR4 and higher biological activity (80, 81). Enterobacteriaceae-derived LPS, which is part of the high-affinity group, activates macrophages at picogram concentrations, making it one of the most potent innate immune activators (4). However, excessive systemic LPS through intravascular administration can induce a cytokine storm characterized by the release of interleukin-6, IFN-γ, and TNF-α, thereby leading to endotoxemia (82).

Symbiotic Gram-negative bacteria inhabit mucosal surfaces, such as the gastrointestinal tract, skin, and oral cavity, releasing LPS into the environment as extracellular vesicles, also known as outer membrane vesicles. In these contexts, LPS contributes more to health maintenance than inflammation. Previous studies have shown that TLR4-deficient mice exhibit increased susceptibility to allergic reactions, reduced macrophage activity, impaired intestinal motility, and decreased neuronal survival, highlighting the role of LPS-TLR4 signaling in maintaining innate immune homeostasis (67, 83, 84). Oral administration of LPS enhances macrophage phagocytic activity without inducing systemic inflammation and confers neuroprotective effects by transforming microglia into a neuroprotective phenotype (65, 85). Thus, LPS can safely regulate innate immunity when administered orally or through mucosal routes.

The “hygiene hypothesis” posits that improved sanitation in urban environments reduces exposure to microbial components, such as LPS, leading to an increased prevalence of allergic diseases (86). This has been linked to an imbalance in T-helper cell responses, with a dominance of Th2 over Th1 immunity (83, 87). Children with gut microbiota dominated by low-LPS-producing *Bacteroides* have been reported to be more prone to allergies than those with high-LPS-producing Enterobacteriaceae (84). These findings indicate that variations in gut microbiota-derived LPS influence allergy susceptibility and can be mitigated by dietary interventions. As mentioned above, LPS can be broadly divided into two types. One is LPS represented by the genus Gammaproteobacteria, which is a strong TLR4 stimulator. The second is LPS represented by the genus Bacteroides, which is a weaker TLR4 stimulator than the former. The difference between these LPS is mainly due to differences in their affinity for TLR4 (88). Therefore, we believe that the difference in function is due to quantitative issues with the LPS functioning, and not due to differences in function between LPS types.

Furthermore, the widespread use of antibiotics, although pivotal in infection control, has disrupted the gut microbiota composition, leading to a reduction in Gram-negative bacterial populations and associated LPS signaling (89, 90). When mice are given antibiotics orally, the density and diversity of intestinal bacteria in their feces decreases by approximately one-tenth or more (89), and the amount of antimicrobial peptide (RegIIIγ) secreted by Paneth cells in the small intestine decreases by one-fifth (90). This antimicrobial peptide suppresses the growth of vancomycin-resistant enterococci (VRE) among intestinal bacteria, and although VRE grows when antibiotics are administered, the growth of VRE is also suppressed by oral administration of LPS (90). Disturbances to the intestinal flora caused by antibiotics are particularly concerning in early childhood. Exposure to antibiotics in the neonatal period has been shown to increase the odds of developing allergies by 2-3 times when assessed between the ages of 2 and adulthood (91), suggesting that it is associated with an increased risk of allergic diseases in later life. Therefore, oral administration of LPS has been suggested as a potential countermeasure to restore innate immune function in such scenarios (90).

With the increasing prevalence of allergic diseases, particularly in urban settings, maintaining adequate environmental exposure to LPS is essential for immune homeostasis. Additionally, developing safe and effective technologies to modulate LPS levels is urgently needed to address these urban-associated diseases. In such cases, it is thought that the LPS of the genus Gammaproteobacteria, which acts in small quantities, is more effective (92).

## 6 Safety of orally and transdermally administered LPS

LPS is routinely ingested through the intestinal mucosa and contributes to health maintenance. However, many biologists do not accept the notion that LPS is an inflammatory substance and sensibly useful. The intravascular administration of LPS induces an extremely strong systemic cytokine storm. The maximum-tolerated dose of the intravenous administration of LPS in humans is as low as 4 ng/kg, and it is an extremely strong inflammatory agent (4). Given that roughly half of the symbiotic bacteria present in the mucous membranes of the intestinal tract, oral cavity, airways, and skin are Gram-negative bacteria, which constantly release outer membrane vesicles, including LPS, it is not surprising that LPS is a substance that hardly induces inflammation in the oral cavity and skin.

Our research has shown that humans are likely to consume LPS daily. Many plants, including vegetables and cereals, have been found to contain more than 10 µg/g of LPS (93, 94). The LPS content in brown rice reaches 10 µg/g, and plant-derived LPS may be involved in maintaining health through daily consumption (6). The levels of LPS in 414 herbal extracts were measured and reported to range from ≥10 ng/g to >100 µg/g (overall mean value 17.4 µg/g). Although the origin of LPS in plants is unknown, it is believed to be derived from Gram-negative bacteria that are in symbiosis with plants in the rhizosphere, where plant hairy roots and soil composition interact with each other, and bacterial density increases by tens to hundreds of times (95). Many symbiotic bacteria in the rhizosphere have been reported to exhibit beneficial effects on plant growth, such as nitrogen fixation, solubilization of insoluble phosphates, and penetration into the plant interior for infection control (96). Additionally, many Enterobacteriaceae bacteria, such as *Escherichia coli*, *Salmonella*, and *Pantoea*, possess LPS, which activates innate immunity in minute quantities (10). Therefore, LPS can be ingested orally in large quantities on a daily basis, and it is considered safe. In fact, when LPS derived from *Pantoea agglomerans*, which is symbiotic with many plants, such as wheat and rice, was administered at a dose of 4500 mg/kg body weight/day for at least 90 days, no adverse effects were observed in terms of clinical symptoms, body weight, food intake, or clinical pathology, and no macroscopic or microscopic findings related to the test substance were observed (97, 98). The LPS derived from *Pantoea agglomerans* has already received Generally Recognized as Safe status, and it has been granted New Dietary Ingredient certifications by the US Food and Drug Administration as an active nutritional ingredient, ensuring its safety.

One concern about the oral administration of LPS, which belongs to the genus Gammaproteobacteria and activates macrophages in small quantities, is that endotoxemia caused by LPS absorbed through the intestinal tract may exacerbate diabetes and atherosclerosis (99, 100). These studies regard high blood LPS levels in mice fed a high-fat diet as endotoxemia. However, this increase in blood LPS levels is known to be due to LPS being absorbed from the intestinal tract together with fat and being bound to lipoprotein particles (chylomicrons) that transport fat in the blood (101). Additionally, LPS bound to chylomicrons is thought to be excreted from the body without causing inflammation (101). Therefore, even though high-fat diets are related to increased blood LPS levels, it cannot be determined as the cause. Furthermore, a previous study (99) introduced a continuous LPS infusion model using a subcutaneous infusion pump as a demonstration experiment to show that high-fat diet-induced endotoxemia exacerbates diabetes. Injected LPS differs significantly from LPS in the blood after oral intake. As mentioned previously, it causes systemic inflammation in minute quantities. In other words, this continuous LPS administration model leads to the worsening of diabetes due to systemic chronic inflammation and has a different mechanism of action from oral LPS administration.

Oral administration of LPS derived from *P. agglomerans*, which belongs to the genus Gammaproteobacteria, has demonstrated a protective effect against diabetes in a mouse model of obese diabetes (KK-Ay) (102), which is contrary to common knowledge. This discrepancy is attributed to the recognition of endotoxemia as a high blood LPS level in mice fed a high-fat diet. However, the increase in blood LPS levels associated with a high-fat diet is based on the fact that LPS is associated with lipoprotein particles (chylomicrons) that are absorbed with fat and transport fat in the blood (101).

LPS has been reported to induce inflammation in certain pathological conditions in the leaky gut, which is characterized by loss of intestinal barrier integrity (99, 100). However, in a mouse model of ulcerative colitis with loss of the intestinal barrier, symptoms have been reported to exacerbate in mice lacking TLR4 of the LPS receptor, and removal of the intestinal microbiota by antibiotic cocktail administration exacerbates ulcerative colitis symptoms, whereas oral LPS administration improves symptoms (103). In other words, LPS in the intestinal tract suppresses enteritis. These findings indicate that the current common knowledge that LPS is responsible for chronic inflammation caused by a leaky gut is open to reconsideration.

## 7 Disease prevention and treatment effects of oral administration of LPS

As discussed thus far, lipopolysaccharides (LPS) derived from symbiotic bacteria present in the environment or on mucosal surfaces are believed to exert beneficial effects on the innate immune system, primarily through macrophage activation, thereby contributing to the maintenance of individual health. Specifically, LPS is thought to play a crucial role in sustaining and enhancing self-healing ability. However, in the modern era, reduced environmental exposure to LPS and the widespread use of antibiotics have led to a situation in which the intentional intake of LPS via mucosal surfaces is actively promoted. Several studies have investigated the role of oral administration of LPS in activating tissue macrophages and inducing prevention and treatment effects (6, 8, 104–106).

The mechanism of action of orally administered LPS leading to self-healing remains unclear. However, the innate immune system may increase its responsiveness to foreign body elimination through epigenetic regulation triggered by activation. The potentiating effect of BCG on the secondary stimulation response of macrophages, which are cells of the innate immune system, is driven by epigenetic regulation and the acquisition of resistance to SARS-CoV2 (107). Similar epigenetic regulation occurs in the phenomenon where stimulation of the LPS receptor TLR4 enhances NF-κB reactivity and drives innate immune system training (108). Such phenomena by LPS have been reported in self-healing related macrophages (6, 8, 104–106, 109), mast cells (110), microglia (110, 111), neutrophils (112), and monocytes (113).

**Figure 2:**
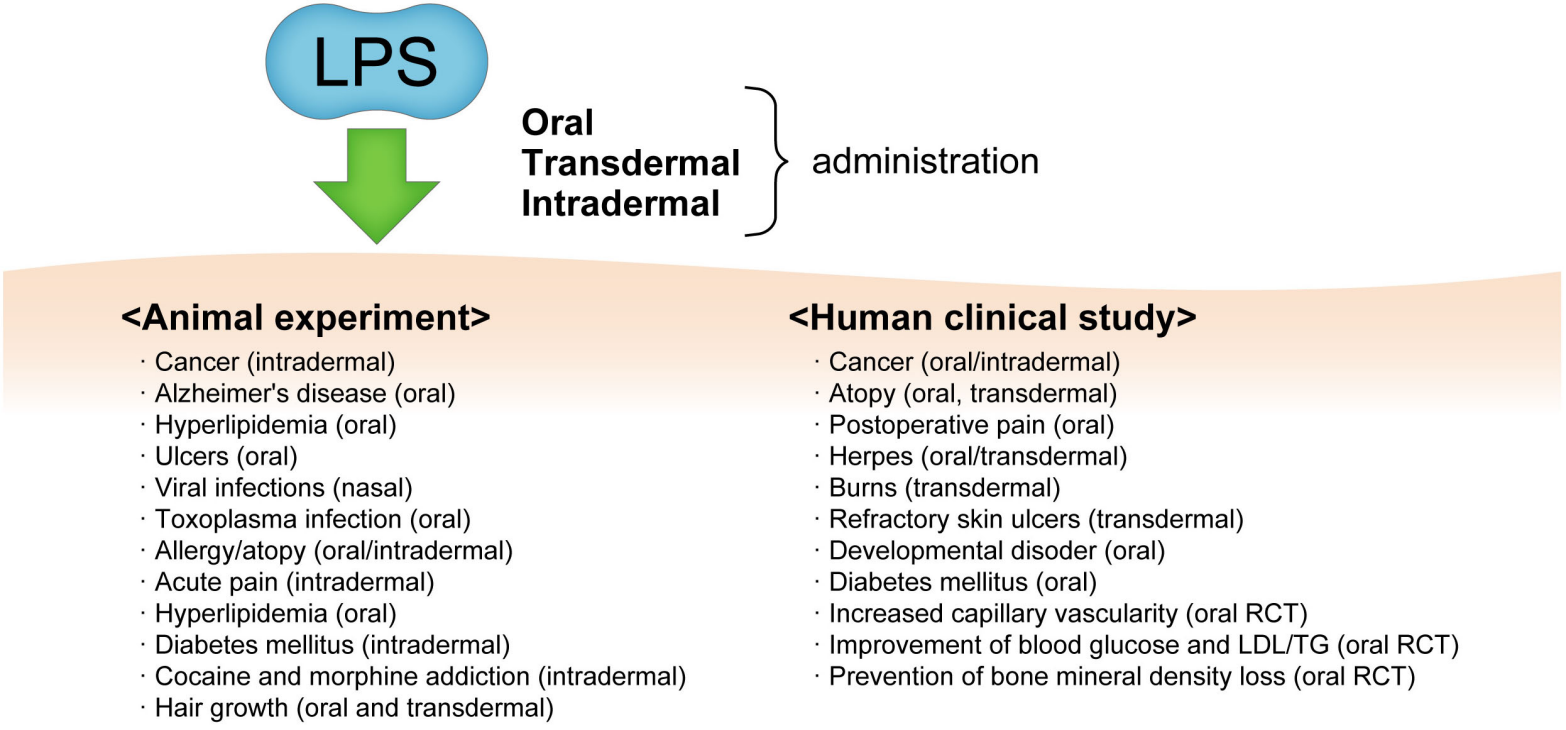
The various diseases and conditions which from oral and/or transdermal administration of LPS and its effects as observed by animal experiments and human clinical studies. RCT, randomized clinical trial; LDL/TG, low-density lipopolyprotein/triglyceride.

The preventive and therapeutic effects of oral administration of LPS on various diseases have been reported in animal and human intervention studies, including reduction or synergistic effect of anti-cancer drugs (114–118), promotion of skin ulcer healing (human intractable skin wounds) (119), promotion of skin ulcer healing (human intractable skin wounds) (119), influenza virus sublingual vaccine adjuvant effect (suppression of influenza infection deaths) (120), suppression of deaths due to toxoplasma infection (121), suppression of plateau reactivity of type I allergy (suppression of IgE-dependent allergic reactions) (8, 122), amelioration of high-fat diet-induced hyperlipidemia (suppression of atherosclerosis) in ApoE-deficient mice (123), suppression of salt-induced blood pressure increase in rats (124), improvement of diabetes and increased adiponectin induction in KK-Ay mice (102), and hair growth (hair growth promotion) (125). Recent studies have reported that the oral administration of LPS promotes the removal of foreign substances by microglia and prevents Alzheimer’s disease (65, 123, 126). Additionally, clinical studies have shown that it is effective in cancer (cancer shrinkage) (127), atopy (improved remission) (128), diabetes (improved markers) (129, 130), capillary dilation (increased) (131), wound healing (accelerated healing of intractable wounds) (132), and developmental disorders (improved) (133) (**Figure 2**).

Kobayashi et al. reported that the oral administration of LPS improved learning function assessed using the Morris water maze test in age-accelerated mice (SAM-P8) fed a high-fat diet as a model of Alzheimer’s disease (123). Additionally, it has been reported to enhance the phagocytic capacity of microglia, the so-called brain-resident macrophages, and induce a significant decrease in amyloid-β levels in the brain and an ameliorating effect on lifestyle-related diseases with significant decreases in diabetes markers, such as HbA1c, oral glucose tolerance test, and fasting blood glucose (134).

Dementia has long been thought to be caused by amyloid-β accumulating in the brain. However, meta-analysis studies have shown that this does not always translate into a therapeutic effect (135). Diabetes in the brain may be a major solitary dementia cause (136). Oral administration of LPS to mice with intracerebroventricular streptozotocin-induced diabetic dementia in the brain has been found to improve learning and memory performance and alter gene expression patterns in the microglia of brain tissue macrophages, exhibiting anti-inflammatory, tissue repair, and neuroprotective effects (65). Furthermore, in this model, learning and memory deficits improved when oral administration of LPS was initiated after cognitive decline, indicating a possible therapeutic effect (137).

Oral administration of LPS has been shown to reduce memory impairment due to dementia in the aging model and diabetes in the brain model. The mechanism underlying this effect involves the removal and repair of damaged neurons by microglia. These findings indicate that oral administration of LPS may be a therapeutic agent that, unlike conventional dementia drugs, repairs and regenerates brain damage via tissue macrophages to exert preventive and therapeutic effects.

Oral and nasal administration of LPS also plays a role in preventing infectious diseases. When mice were given free drinking administration of LPS and infected with Salmonella via intraperitoneal injection, the LPS treatment significantly prolonged survival (6). In addition, when mice were given LPS intranasally 3 to 1/2 days before infection with influenza virus, it significantly prevented death from influenza (138). Furthermore, sublingual administration of LPS significantly increases the production of immunoglobulin A in the mucosa of the influenza vaccine, and reduces mortality (120). Since influenza is a respiratory tract infection that is neutralized by immunoglobulin A in the respiratory tract mucus before it enters the body, it is possible to develop an effective sublingual vaccine.

Chronic inflammation caused by obesity is a significant factor in many lifestyle-related diseases, such as dementia, atherosclerosis, diabetes, and cancer. Oral administration of LPS has been reported to have the ability to prevent obesity. Yamamoto et al. reported that LPS intake in KK-Ay mice improved fasting blood glucose, insulin sensitivity, and HbA1c, and notably, in adipose tissue, adiponectin protein and gene expression significantly increased. However, no changes in inflammatory cytokines were observed (102). Adiponectin is an anti-inflammatory, anti-obesity cytokine produced by adipocytes (139), a phenomenon that is opposite to the fact that LPS is considered a diabetes-increasing factor (140, 141) and awaits further analysis.

The health-maintaining effects of LPS exposure may be analogous to the hormesis effects of radiation exposure. High-dose radiation is harmful to health, whereas low-dose radiation may contribute to health maintenance (142). In this light, the human body may possess a mechanism by which innate immune activation by environmental stimuli at levels where the response is temporarily undetectable is responsible for health maintenance, and the regulation of innate immune activation by oral administration of LPS may be one such system.

## 8 Effects of transdermal administration of LPS

The skin is considered the second most important interface for interaction with symbiotic bacteria from the external environment after the intestinal tract. This section explores the role of lipopolysaccharides (LPS) in maintaining and enhancing self-healing ability, primarily through macrophage activation, when applied to the skin. LPS exhibits a high affinity for sebum within the stratum corneum. However, the passage of molecules larger than 500 Da, such as LPS, is restricted by tight junctions in the granular layer of the lower epidermis (143). Despite this barrier, LPS interacts with TLR4 expressed on Langerhans cells. These cells extend their dendrites beyond tight junctions, facilitating the binding of LPS to TLR4 (144). Langerhans cells, historically categorized as dendritic cells, have been reclassified as macrophages specific to skin tissue (145). These cells exhibit functional changes upon stimulation with LPS, including the downregulation of the allergic chemokine thymus-activation-regulated chemokine. This suppression of thymus-activation-regulated chemokine expression is associated with a reduction in allergic responses, highlighting the potential role of LPS-stimulated Langerhans cells in mitigating allergic inflammation (144). In the skin, TLR4 is expressed not only on Langerhans cells but also transiently on damaged epidermal keratinocytes within 24 h of injury. Stimulation with LPS has been reported to enhance the wound-healing capacity of epidermal cells (146). *In vitro* studies using human epidermal keratinocyte models (HaCaT cells) have shown that LPS promotes cell migration, a critical step in wound closure (147). Furthermore, similar effects of TLR4 activation by LPS have been observed in other epithelial tissues. Lung and corneal epithelial cells upregulate TLR4 expression in response to physical stimuli. LPS stimulation in these cells has been reported to promote wound healing, whereas blocking TLR4 signaling with specific inhibitors or antibodies delays healing (146, 147). These findings indicate that LPS directly influences epidermal and epithelial cells by activating TLR4, thereby enhancing their wound-healing properties.

LPS has been reported in cell studies to interact with keratinocytes at the apical side of tight junctions, inducing antimicrobial peptide production and enhancing the epidermal barrier by upregulating the expression of filaggrin and claudin-1, which are key components associated with tight junction integrity (148). In human intervention studies, LPS derived from *Pantoea vagans* has demonstrated additional benefits, including skin hypersensitivity suppression, increased stratum corneum hydration, and reduced transepidermal water loss, in individuals with sensitive skin (149). Similarly, a double-blind human intervention study has reported that applying LPS derived from*P. agglomerans* to the skin significantly reduces the itching of mild atopic dermatitis and improves the skin condition (128). These findings indicate that transdermal application of LPS contributes to skin homeostasis by preventing and reducing skin disorders. Mechanistically, LPS enhances skin barrier function and regulates moisture balance, indicating its potential as a therapeutic agent for managing sensitive or compromised skin conditions. Additionally, activation of skin tissue by LPS may help prevent skin aging. It has been shown in cell models and ex-vivo models using human skin that senescent cells in the dermis are phagocytosed and removed via receptors such as STAB1 (phosphatidylserine receptor), CD36, and advanced glycation end-product receptor (18, 150). Macrophages activated by LPS exhibit increased phagocytic activity in co-culture condition, facilitating the removal of senescent cells and foreign substances (151). *In vitro* studies have shown that LPS-stimulated macrophages modulate the properties of primary human fibroblasts, enhancing cell proliferation, hyaluronic acid synthesis, and elastin expression. These findings indicate that transdermal administration of LPS activates dermal macrophages, thereby contributing to the removal of senescent cells and the prevention of skin aging. Furthermore, a recent study reported that the thinned epidermis of aging mice regained thickness compared with that of younger mice following LPS application (122).

Consequently, transdermal application of LPS supports skin homeostasis by improving epidermal barrier function, alleviating skin sensitivity, and promoting dermal repair mechanisms, resulting in enhancing self-healing ability. Additionally, LPS can prevent skin aging by activating macrophages, enhancing fibroblast function, and clearing senescent cells. These findings underscore the self-healing potential of LPS in addressing skin disorders and age-related changes in skin integrity.

## 9 Discussion

In this review, we introduced the concept of the macrophage network as an innate immune mechanism responsible for health maintenance and natural healing. Although there have been many reports of LPS showing toxicity depending on the type and administration methods (152, 153), we will outline the latest findings and applications regarding the prevention and treatment effects of various diseases through oral and transdermal administration of LPS.

Humans are constantly exposed to the risk of infection by viral, bacterial, and other pathogens, such as coronaviruses that have emerged in the last few years. The human body eliminates emerging infectious pathogens mainly through innate immune function. However, if the body is continuously exposed to excessive stress levels, the innate immune system is weakened, the organism’s homeostasis is severely impaired, and the body loses its ability to resist emerging infectious pathogens (6, 154). Therefore, establishing measures to prevent the weakening of innate immunity is important (155–157). Further advances in our understanding of self-healing based on innate immune function can contribute to the development of effective disease prevention and treatment strategies, such as oral and transdermal administration of LPS.

Determining the state of innate immune function is necessary for assessing health and innate healing. To this end, a comprehensive index of innate immunity should be established, test methods should be identified, and assessment criteria should be developed. However, because innate immune responses are diverse and heterogeneous, focusing on a single group of cells is insufficient. The development of non-invasive, user-friendly methods to assess innate immune responses is an important challenge. Despite being challenging, further efforts should be made to design effective devices for the routine monitoring of innate immune function.

Even healthy individuals may be carriers of hidden premorbid conditions, such as pre-diabetes, hypertension, and dyslipidemia (124, 155). These diseases are associated with reduced innate immune function and impaired self-healing. Using pharmaceuticals to treat these supposedly healthy but disease-prevalent conditions is highly problematic. Therefore, proper functioning of the innate immune system is essential for achieving optimal health. Furthermore, in diseased individuals, restoring the innate immune function can maximize the effectiveness of medicinal products. The significance of maintaining innate immune function and using LPS is expected to get wider recognition in the coming years.
